# Dynamic Zinc Accumulation and Contributions of Pre- and/or Post-Silking Zinc Uptake to Grain Zinc of Maize as Affected by Nitrogen Supply

**DOI:** 10.3389/fpls.2019.01203

**Published:** 2019-10-03

**Authors:** Yan-Fang Xue, Shan-Chao Yue, Dun-Yi Liu, Wei Zhang, Xin-Ping Chen, Chun-Qin Zou

**Affiliations:** ^1^Key Laboratory of Plant-Soil Interactions, Ministry of Education, Center for Resources, Environment and Food Security, China Agricultural University, Beijing, China; ^2^Maize Research Institute, Shandong Academy of Agricultural Sciences/National Engineering Laboratory of Wheat and Maize/Key Laboratory of Biology and Genetic Improvement of Maize in Northern Yellow-huai River Plain, Ministry of Agriculture, Jinan, China; ^3^State Key Laboratory of Soil Erosion and Dryland Farming on the Loess Plateau, Northwest A&F University, Yangling, China; ^4^College of Resources and Environment, Southwest University, Chongqing, China

**Keywords:** nitrogen supply, dynamic Zn accumulation, Zn uptake, Zn remobilization, summer maize

## Abstract

Nitrogen (N) supply could improve the grain yield of maize, which is of great importance to provide calories and nutrients in the diets of both humans and animals. Field experiments were conducted in 2009 and 2010 to investigate dynamic zinc (Zn) accumulation and the pre-silking and post-silking Zn uptake and their contributions to grain Zn accumulation of maize with different N supply under field conditions. Results showed that only 1.2% to 39.4% of grain Zn accumulation derived from pre-silking Zn uptake, with Zn remobilization being negatively affected by increasing N supply. However, post-silking Zn uptake (0.8–2.3 mg plant^–1^) and its substantial contribution to grain Zn accumulation (60.6%–98.8%) were progressively enhanced with the increasing N supply. Furthermore, grain Zn concentration was positively associated with grain N concentration (r = 0.752***), post-silking N uptake (r = 0.695***), and post-silking Zn uptake (r = 738***). A significant positive relationship was also found between post-silking uptake of N and Zn (*r* = 0.775***). These results suggest that N nutrition is a critical factor for shoot Zn uptake and its allocation to maize grain. Dry weight, and N and Zn concentration of grain and straw were significantly enhanced with the increasing N from “no N” to “optimal N” supply (150 kg N ha^−1^ in 2009 and 105 kg N ha^−1^ in 2010), but further increasing N supply (250 kg N ha^−1^) generally resulted in a non-significant increase in both cropping seasons. During the grain development, N supply also generally tended to improve grain N and Zn concentrations, but decrease phosphorus (P) concentration and the molar ratio of P to Zn compared with null N application. These results suggest that grain Zn accumulation mainly originates from post-silking Zn uptake. Applying N at optimal rates ensures better shoot Zn nutrition and contributes to post-silking Zn uptake, maintaining higher grain Zn availability by decreasing the molar ratio of P to Zn, and resulting in benefits to human nutrition.

## Introduction

Zinc (Zn) deficiency represents a globally common and important nutritional disorder, affecting one third of people worldwide, mainly in developing countries where cereals constitute the major part of the diet (Hotz and Brown, 2004; Welch and Graham, 2004). Maize (*Zea mays* L.) is not only a globally important crop mainly utilized as feed but also as food and raw material for diverse industrial applications. It is estimated that maize, as a dietary staple for more than 200 million people, provides around 15% of the world’s protein and 20% of the world’s calories (Nuss and Tanumihardjo, 2010). Thus, increasing Zn levels in maize grain could deliver more Zn to people whose diet relies directly or indirectly on maize-derived food to mitigate Zn deficiency in human beings.

In China, maize accounts for around 39% of Chinese cereal production, and China is responsible for 22% of global maize output from 2012 to 2014 (FAO, 2017). To meet the needs of the developing population in the world (for example the Chinese population is expected to peak at 1.5 billion in 2033), grain production must increase by at least 35% during the next 20 years (Zhang, 2011). Pursuing high grain yields in China has been the top priority in policy. However, the agricultural system in China relies heavily on high-to-excessive inputs. For example, nitrogen (N) application rate in China is around four times higher than global application rates (305 kg N ha^−1^ year^−1^ vs 74 kg N ha^−1^ year^−1^), whereas N use efficiency (the fraction of N input harvested as product) is only 0.25 compared with 0.42 globally, and 0.65 in North America (Zhang et al., 2015a). Over-application of N fertilizer has caused widespread soil acidification, devastating water pollution, and excessive greenhouse gas emissions (Diaz and Rosenberg, 2008; Guo et al., 2010; Zhang et al., 2016).

Many previous studies have shown that adequate N supply could effectively enhance grain Zn concentration of wheat grown under both field and glasshouse conditions (Kutman et al., 2010; Shi et al., 2010; Gooding et al., 2012; Xia et al., 2018). In contrast, several studies have reported that increasing N application slightly decreased the grain Zn concentration of maize (Feil et al., 2005; Losak et al., 2011; Chen et al., 2016). Other research has identified an increase in grain Zn concentration of maize with the increasing N supply (Oktem et al., 2010; Grujcic et al., 2018), although to a lesser extent than found in wheat (Kutman et al., 2010; Xue et al., 2012). Strategies to improve N management and the development of related policies to achieve this in crop production and environmental protection are currently high on the political agenda in China. Our previous studies showed that optimal N management (achieved by reasonably decreasing N input) did not result in any significant decrease in grain yield or Zn concentration of shoot and grain of wheat (Xue et al., 2012; Xue et al., 2014a). However, how the optimization of N management (by decreasing excessive N supply rate) affects maize Zn nutrition and its allocation into grain under field conditions needs to be further evaluated.

It is known that there are two main sources of Zn accumulation in cereal grain: uptake that is concurrent with grain filling and that resulting from post-anthesis remobilization from the Zn stored in source tissues (Waters et al., 2009; Hegelund et al., 2012). Previous studies reported that the grain Zn provided by remobilization of the pre-anthesis Zn stores accounted for 58% to 60% (Dang et al., 2010) and 67% to 100% (Xue et al., 2012) of total wheat grain Zn under field conditions. However, very little is known about how pre- and post-silking Zn uptake and their contributions to grain Zn accumulation of maize are affected by N supply.

A previous study has reported the dynamics of grain Zn accumulation during the maize grain development, showing that grain Zn concentration decreased to a large extent in the late stage of milky maturation and thereafter fluctuated slightly before full grain maturity (Horvatić et al., 1998). The same authors also found that the molar ratios of phytic acid (PA) to Zn tended to increase during grain development. In the maturing grains of maize, the total phosphorus (P) could be an indicator of phytate because 80% of the P was in the form of phytate (Raboy, 1997). Hence, identifying the relationships between P and Zn concentrations in maize grain, as affected by N application rates during grain development, could be a tractable method to estimate Zn availability in maize grain.

The aims of this study were to determine how the different N application rates affected the i) the temporal dynamics of Zn accumulation in the aboveground tissues, and grain of maize, and grain Zn availability (estimated as the molar ratio of P to Zn), and ii) the pre-silking and post-silking Zn uptake by maize, and their relative contributions to Zn accumulation in grain under field conditions.

## Materials and Methods

### Field Locations

The field experiments were conducted in 2009 and 2010, as part of a long-term N-fertilization experiment initiated in 2007 in Quzhou County (36.9°N, 115.0°E), Hebei province, China. The soil was calcareous alluvial soil with the following characteristics of the top 30 cm soil layers: pH of 8.3 (1:2.5 w/v in water), total N of 0.83 g kg^–1^, Olsen-P of 7.2 mg kg^–1^, available potassium of 125 mg kg^–1^, organic matter of 1.42%, the bulk density of 1.36 g cm^–3^. The soil DTPA-extractable Zn concentrations before sowing were 2.3 mg kg^–1^ in 2009 and 2.8 mg kg^–1^ in 2010. Daily mean temperature, solar radiation, and precipitation during the two growing seasons were presented in **Figure S1**. Accumulated growing degree days with a base temperature of 10°C during the two growing seasons were 1,737°C in 2009 and 1,681°C in 2010 and total precipitation was 339 mm in 2009 and 277 mm in 2010. In addition, 50 and 70 mm of irrigation were applied in 2009 and 2010, respectively after sowing.

### Experimental Design

A maize cultivar (*Zea mays* L., cv. Zhengdan958) was planted in two cropping seasons. Maize was planted without tillage after harvest of winter wheat in mid-June, with a row spacing of 60 cm. Maize was harvested at the beginning of October, and the average harvest densities were 7.7 and 7.8 plants m^–2^ in 2009 and 2010, respectively. A randomized complete block experimental design was used with four replicate plots (300 m^2^ plot^–1^) in 2009 and with three replicate plots (100 m^2^ plot^–1^) in 2010. Three N application rates, as urea, were applied as follows: no N application (recorded as N0, control), optimal N application (150 kg N ha^–1^ in 2009 and 105 kg N ha^–1^ in 2010, recorded as N1) and the farmer’s N practice (a preplanting application of 100 kg N ha^−1^ and the topdressing of 150 kg N ha^−1^ at 6-leaf stage, recorded as N2). The optimal N application was determined following the assignment of in-season root-zone N management strategies (Cui et al., 2008). The growth of maize was divided into three periods, depending on plant N uptake and farmers’ fertilization practices: i) from planting to the V6 (six-leaf) stage, ii) from the V6 to the V10 (ten-leaf) stage, and iii) from the V10 to harvest stage. At the V3 (three-leaf) stage, 45 kg N ha^–1^ was applied. N1 treatment was determined using the V6 to V10 stages and the V10 to harvest stages by subtracting measured soil nitrate-N content in the root zone (0–60 and 0–90 cm for the two growth stages, respectively) from the target N value. The target values for each successive growth period, estimated based on the target yield and crop N uptake, were 50, 120, and 190 kg N ha^–1^, respectively. If calculated N1 treatment was less than 30 kg N ha^−1^, we applied 30 kg N ha^−1^ of N fertilizer for the N1 treatment. Details of N target value, soil nitrate-N content in the root layers, and N application rates for different growth stages and years were listed in **Table S1**. In addition, 45 kg of P_2_O_5_ ha^–1^ as superphosphate, 90 kg of K_2_O ha^–1^ as potassium chloride and 30 kg of ZnSO_4_.7H_2_O ha^–1^ were applied deep (10 cm) into the soil with a furrowing machine at the V3 stage, accompanied by the application of urea.

### Sampling and Nutrient Analysis

In 2009, six-plant samples were collected at V6, V12 (twelve-leaf), R1 (silk emerging), R3 (milk stage), and R6 (physiological maturity) growth stages. The R6 sample was separated into grain and straw samples. In 2010, six-plant samples were collected at growth stages R1, R3, and R6 and each divided into leaves, stems, cobs, and grains. In addition, to study the accumulation of N, Zn, and P during the seed development, as affected by the N application rates, seed sampling was conducted in 2009 every 5–7 days after silking (DAS) until ripening. Soil samples were taken from 0 to 90 cm depth in each plot at R1 and R6 growth stages in both years. Fresh soil samples were extracted with 0.01 mol L^–1^ CaCl_2_ and analyzed for NH_4_^+^–N and NO_3_^–^–N using continuous flow analysis (TRAACS 2000). In this report, the term “shoot” refers to all aboveground tissues of maize plants, including the straw and grain. All samples were rapidly washed with deionized water and then oven-dried at 60°C to 65°C to determine the dry weight. Plant samples were ground with a stainless-steel grinder (RT-02B, Taiwan, China) and digested with HNO_3_–H_2_O_2_ in a microwave accelerated reaction system (CEM, Matthews, NC, USA). The concentrations of Zn and P in the digested solutions were determined by inductively coupled plasma atomic emission spectroscopy (ICP-AES; OPTIMA 3300 DV, Perkin-Elmer, USA). IPE556 grain and IPE883 straw (Wageningen University, the Netherlands) were used as reference materials. Nitrogen concentration in the samples was analyzed by the micro-Kjeldahl procedure after digestion with H_2_SO_4_–H_2_O_2_. All results are expressed on a dry weight basis.

### Calculations and Statistical Analysis

Shoot Zn concentration = the sum of the Zn contents of all aboveground tissues/the sum of the dry weight of all aboveground tissues

Post-anthesis Zn uptake = shoot Zn content at maturity - shoot Zn content at anthesis

Percentage of pre-anthesis Zn accumulation = shoot Zn content at anthesis/shoot Zn content at maturity

Percentage of post-anthesis Zn accumulation = post-anthesis Zn uptake/shoot Zn content at maturity

Zn remobilization to the grain = shoot Zn content at anthesis - straw Zn content at maturity

Zn remobilization efficiency = Zn remobilization/shoot Zn content at anthesis

Share of grain Zn provided by remobilization = Zn remobilization to the grain/grain Zn content at maturity

Share of grain Zn provided by post-anthesis shoot Zn uptake = (grain Zn content at maturity - Zn remobilization)/grain Zn content at maturity

Zn harvest index (ZnHI) = grain Zn content/shoot Zn content at maturity

All the above calculations were reported previously by Xue et al. (2012).

The effects of N fertilization treatments on the dependent variables were determined using one-way ANOVA and SAS software (SAS 8.0, USA). When the effects were significant, treatment means were compared by the least significant difference (LSD) test at P < 0.05. The Pearson correlation procedure was used to evaluate the relationships among the reported parameters.

## Results

### Dry Weight and Zn and N Concentrations of Grain and Straw at Maturity

Dry weight (DW) and N concentration of grain and straw were significantly increased by increasing N rate from N0 to N1, but further increasing N rate from N1 to N2 resulted in a non-significant increase in both cropping seasons. Similar trends were also observed for Zn concentration of grain and straw, with the exception of an increase in grain Zn concentration with increasing N rate from N0 to N2 in 2009 (**Table 1**). Grain Zn concentration was significantly positively correlated with grain N concentration (r = 0.752***) and grain dry weight (r = 0.614**) (**Table 2**). The average harvest index of Zn (ZnHI) was 48.3% and 44.5% and average harvest index of N (NHI) was 67.3% and 64.4% in 2009 and 2010, respectively. These harvest indexes were not affected by different N application rates, cropping years or their interaction (**Table 1**).

**Table 1:** Dry weight of grain and straw, Zn and N concentrations of grain and straw, and grain Zn (ZnHI) and N (NHI) harvest index of summer maize as affected by N application rates in 2009 and 2010, respectively, in the field.

Year	N treatment	Dry weight (g plant^–1^)		Zn concentration (mg kg^-1^)		N concentration (%)			
		Grain	Straw	Grain	Straw	Grain	Straw	ZnHI	NHI
2009	N0	88.3b	82.3b	14.9c	15.2b	1.2b	0.6b	51.9a	69.5a
	N1	131.3a	118.3a	17.1b	21.6a	1.4a	0.7a	46.8a	67.9a
	N2	127.9a	112.6a	18.5a	24.8a	1.3ab	0.8a	46.3a	64.5a
2010	N0	84.3b	74.3b	15.2b	20.3a	1.2b	0.6b	45.8a	67.1a
	N1	120.3a	118.4a	18.8a	25.0a	1.4a	0.8a	43.7a	63.5a
	N2	122.1a	122.0a	19.3a	25.3a	1.5a	0.9a	43.9a	62.6a
Source of variation									
N treatment (N)		***	***	***	*	***	***	ns	ns
Year (Y)		ns	ns	*	ns	ns	*	ns	ns
N*Y		ns	ns	ns	ns	ns	ns	ns	ns

Values followed by different lowercase letters in columns represent significant differences among the different N application rates.

ns indicates no significant difference. *, and *** indicate significant difference at P < 0.05, and < 0.001, respectively.

**Table 2:** Correlative coefficients (r) among grain dry weight (GDW), post-anthesis Zn uptake (Zn_uptake_), post-anthesis N uptake (N_uptake_), grain Zn concentration (GZnC), and grain N concentration (GNC) in 2009 and in 2010 (*n* = 21).

Parameter	GDW	Zn_uptake_	N_uptake_	GZnC
Zn_uptake_	0.684***	–	–	–
N_uptake_	0.714***	0.775***	–	–
GZnC	0.614**	0.738***	0.695***	–
GNC	0.524*	ns	0.476*	0.752***

ns indicates no significant difference. *, **, and *** indicate significant difference at P < 0.05, < 0.01, and < 0.001, respectively.

### Dynamic Dry Weight and N and Zn Uptake and Concentration in Shoot

**Figure 1:**
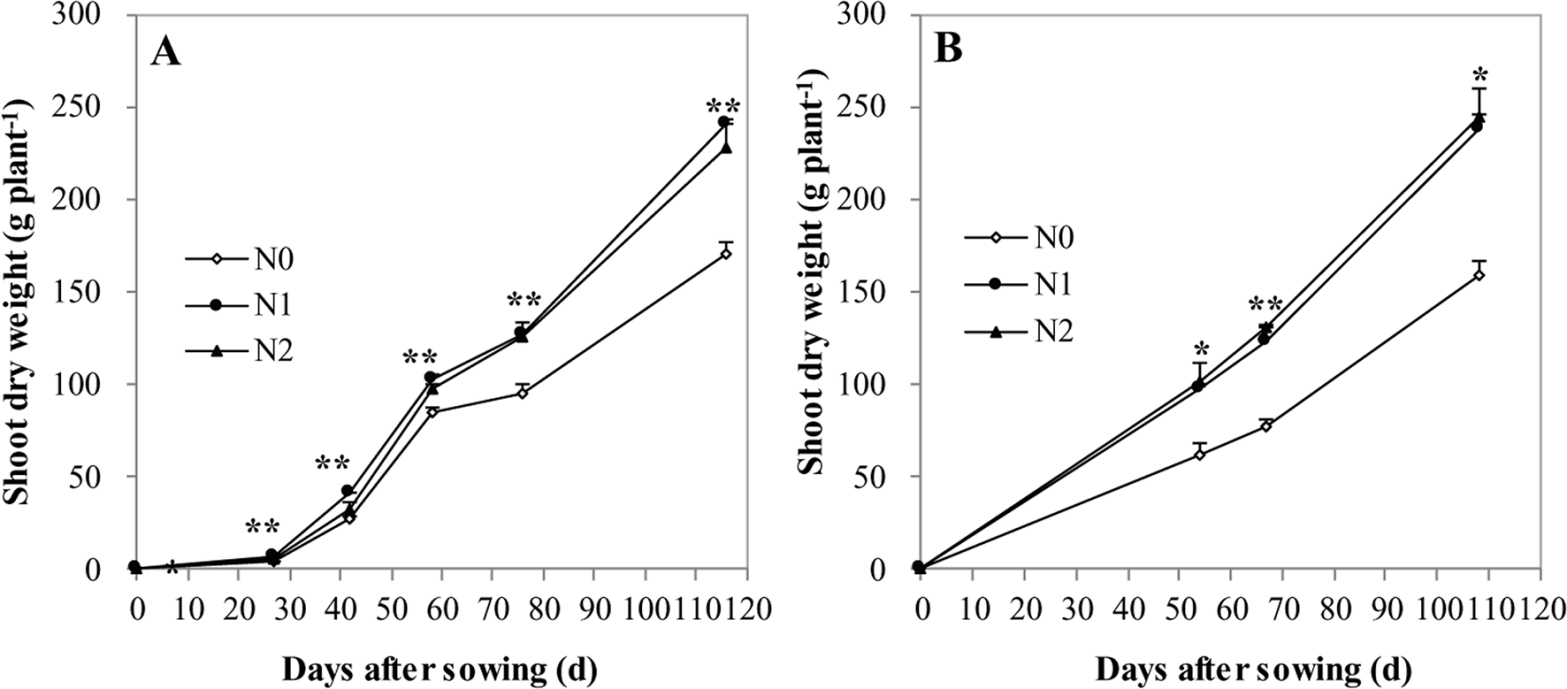
The shoot biomass of summer maize at V6, V12, R1, R3, and R6 growth stages as affected by N application rates in the field in 2009 **(A)** and 2010 **(B)**, respectively. The error bars represent the standard error of the mean (*n* = 4 in 2009 and *n* = 3 in 2010). *, ** indicate significant difference at P < 0.05, < 0.01, respectively.

**Figure 2:**
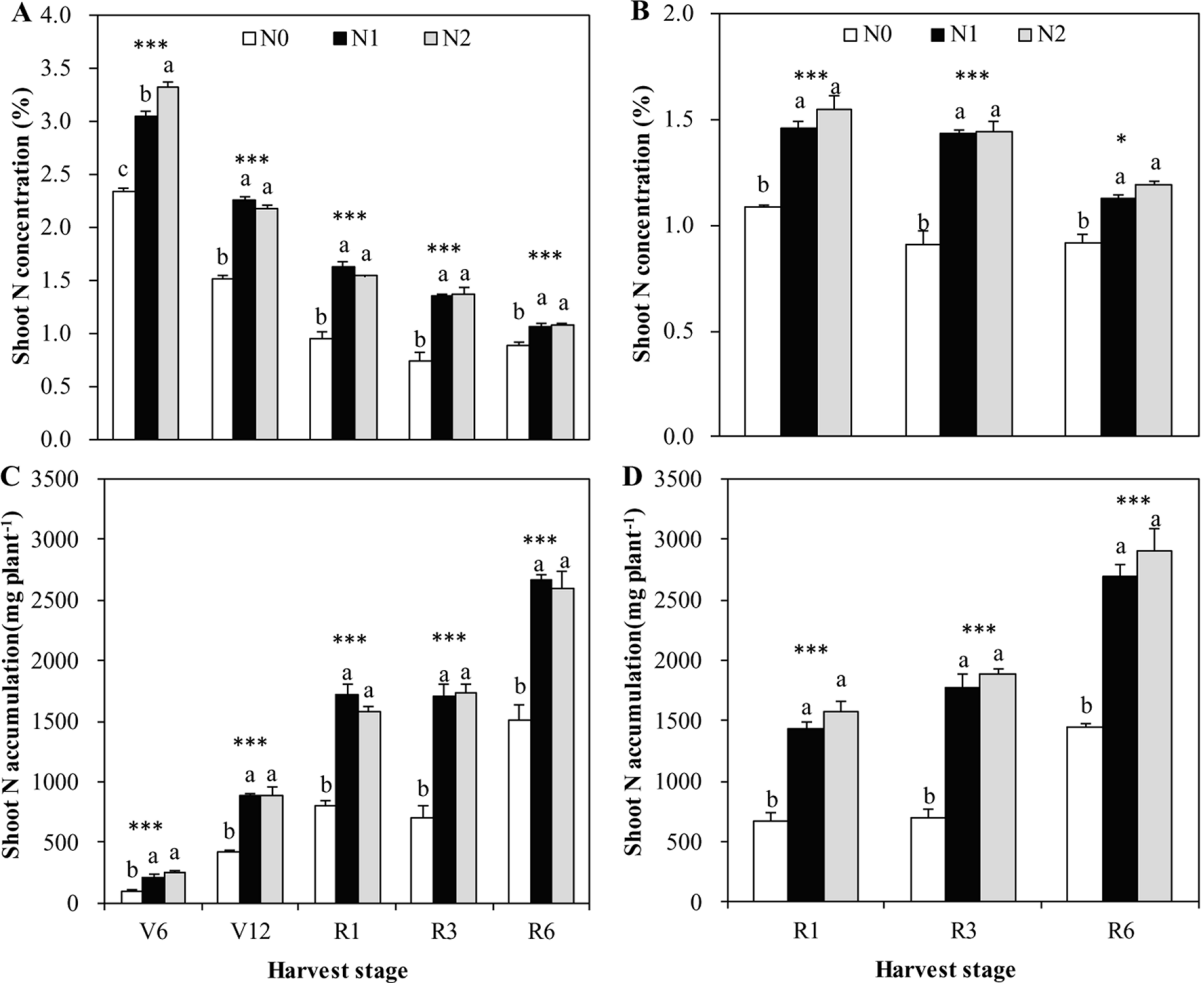
The shoot N concentration and accumulation of summer maize at V6, V12, R1, R3, and R6 growth stages as affected by N application rates in the field in 2009 **(A**, **C)** and 2010 **(B**, **D)**, respectively. The error bars represent the standard error of the mean (*n* = 4 in 2009 and *n* = 3 in 2010). For each stage, bars with same lowercase letters are not significantly different in different N treatments (P < 0.05). ns indicates no significant difference. * and *** indicate significant difference at P < 0.05, and < 0.001, respectively.

**Figure 3:**
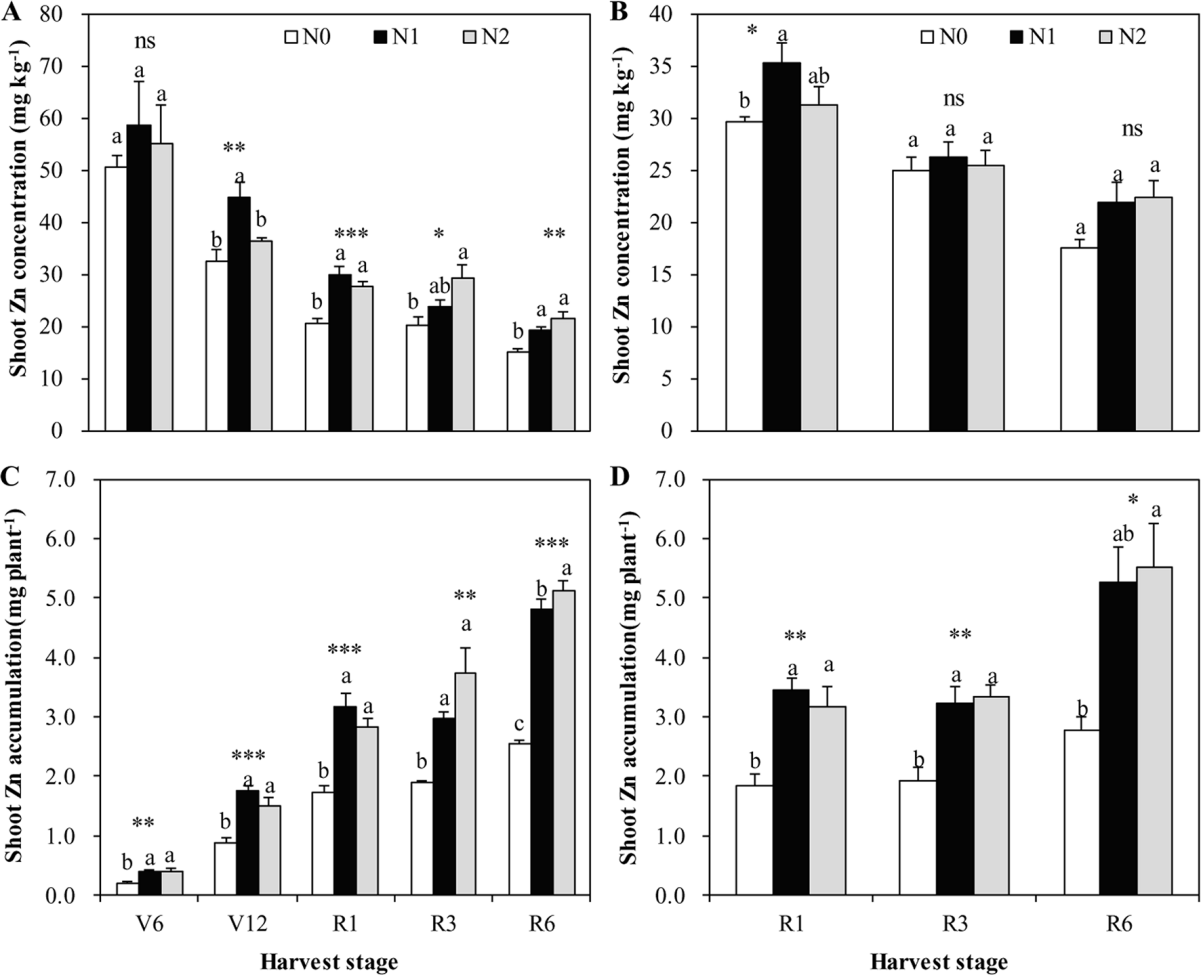
The shoot Zn concentration and accumulation of summer maize at V6, V12, R1, R3, and R6 stages as affected by N application rates in 2009 **(A**, **C)** and 2010 **(B**, **D)**, respectively, in the field. The bars represent the standard error of the mean (*n* = 4 in 2009 and *n* = 3 in 2010). For each stage, bars with same lowercase letters are not significantly different in different N treatments (P < 0.05). ns indicates no significant difference. *, **, and *** indicate significant difference at P < 0.05, < 0.01, and < 0.001, respectively.

Irrespective of N treatments, increasing shoot DW accumulation toward maize maturity resulted in a declined shoot N and Zn concentration, a so-called general dilution effect (**Figures 1**–**3**). The positive effects of N supply on DW and the shoot accumulation of N and Zn were found at all growth stages. The shoot DW significantly increased with increasing N supply from N0 to N1, but further increasing N supply from N1 to N2 resulted in a non-significant increase in both cropping seasons (**Figures 1A, B**). Similar trends were also observed for shoot N concentration and accumulation at all growth stages in both years with the exception that there was a significant difference in shoot N concentration between N1 and N2 at V6 stage in 2009 (**Figure 2**).

Shoot Zn concentration increased with increasing N rate from N0 to N1, with the effects being more pronounced in 2009 than 2010 (**Figure 3**). However, further increases in N rate from N1 to N2 resulted in a non-significant increase in shoot Zn concentration, with the exception that there was a significantly lower shoot Zn concentration in N2 than N1 at V12 stage in 2009 (**Figure 3A**). Shoot Zn accumulation significantly increased with the increasing N rate from N0 to N1 at all growth stages in both years (**Figure 3**). However, further increasing N rate from N1 to N2 resulted in a non-significant increase in shoot Zn accumulation, with the exception that there was a significantly greater shoot Zn accumulation in N2 than N1 at R6 stage in 2009 (**Figure 3C**).

### Shoot Concentration and Accumulation of Zn Response to N Availability

**Figure 4:**
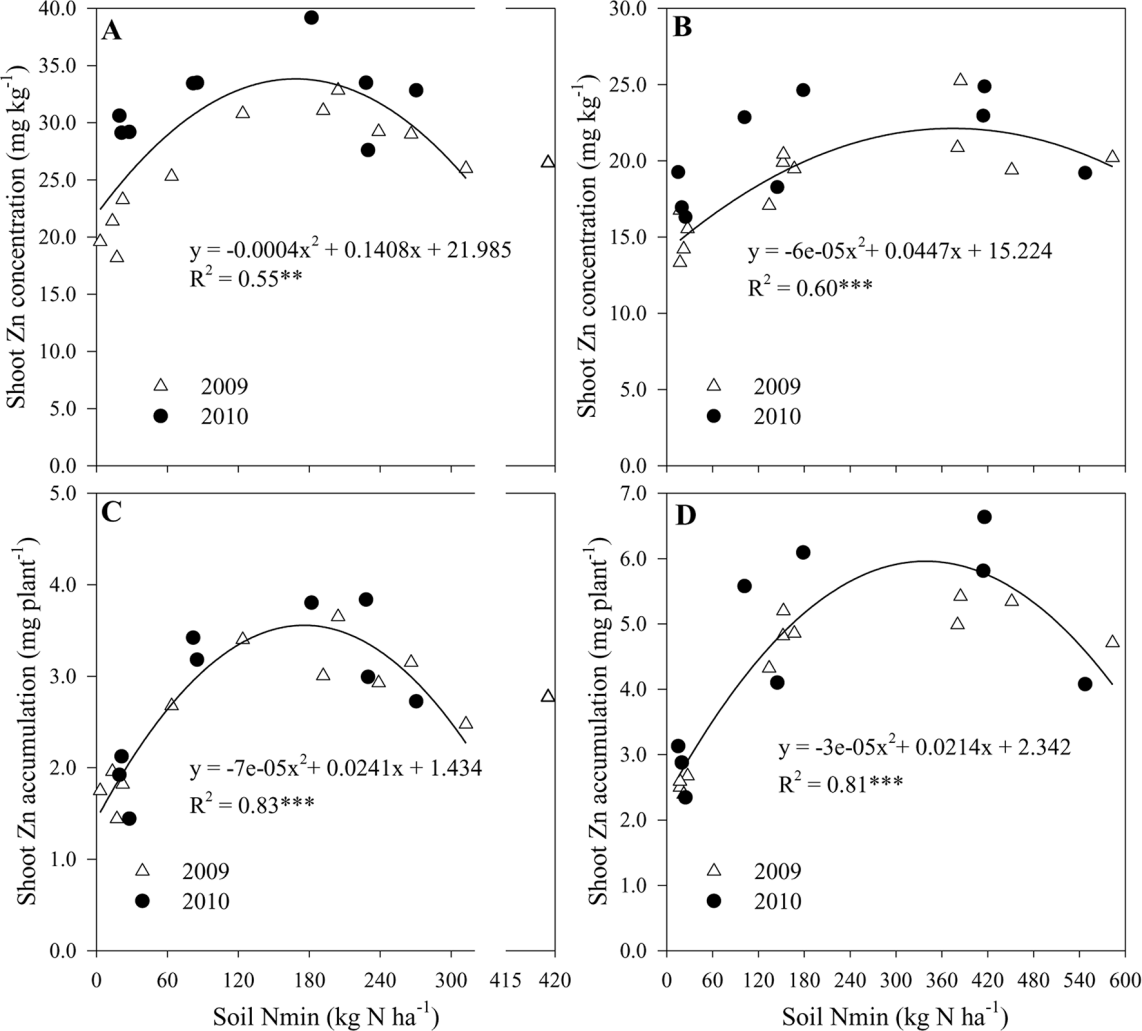
The shoot concentration and accumulation of Zn at R1 **(A**, **C)** and R6 **(B**, **D)** growth stages, respectively as function of an increasing N availability in soil at 0–90 cm in 2009 and 2010. ** and *** indicate significant difference at P < 0.01 and < 0.001, respectively.

**Figure S2** showed that the NO_3_^–^N contents at different soil layers increased with the increasing N supply from N0 to N2 treatment. Furthermore, shoot concentration and accumulation of Zn at R1 and R6 growth stages could be predicted across both years by a quadratic equation based on N availability in soil. The quadratic equation predicts the highest Zn concentration and accumulation in shoots at growth stage R1, with 34.4 mg kg^−1^ and 3.6 mg plant^−1^ predicted when the cumulative Nmin in soil of 0-90 cm was 176 kg N ha^−1^ (**Figures 4A, C**). At maturity, these peak Zn values approached to 22.9 mg kg^−1^ and 6.0 mg plant^−1^ when the cumulative Nmin was 344 and 339 kg N ha^−1^, respectively (**Figures 4B, D**).

### Dynamic Dry Weight and N, P, and Zn Accumulation of Grain During Grain Development

**Figure 5:**
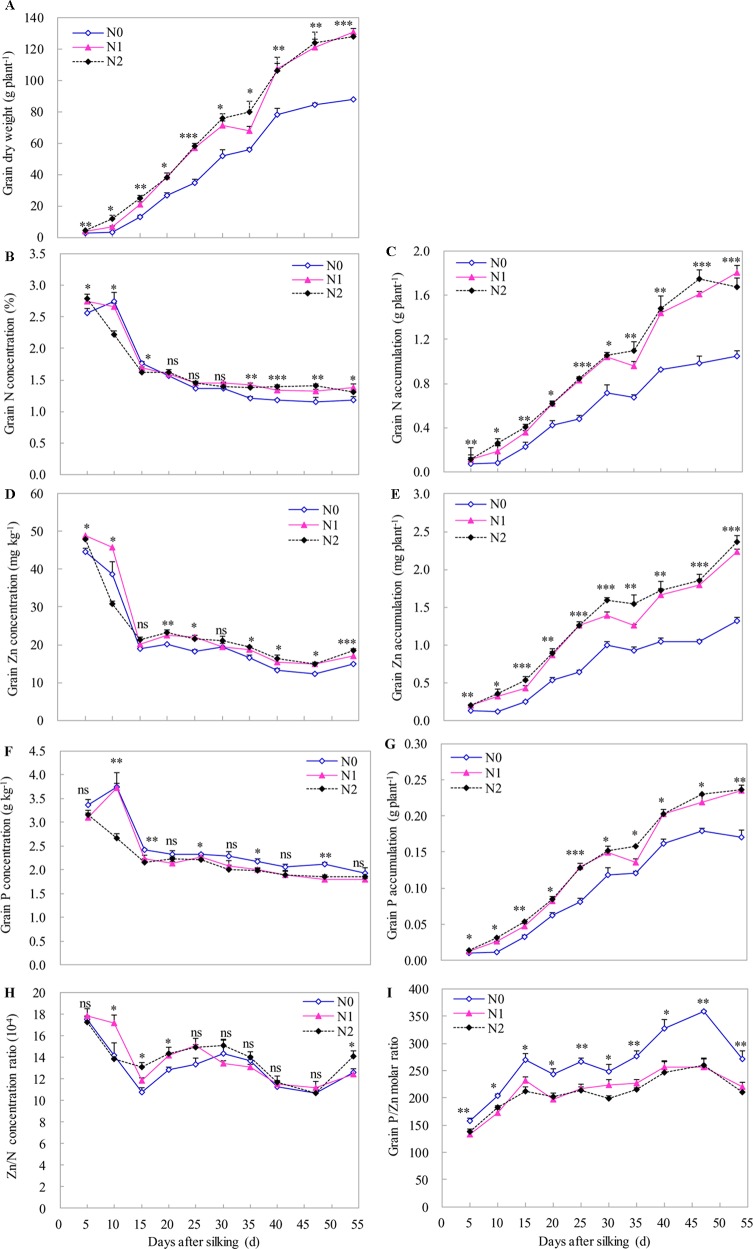
The dynamics of grain dry weight **(A)**, N, Zn and P concentration **(B**, **D**, **F)** and accumulation **(C**, **E**, **G)**, the ratios of Zn to N accumulation **(H)** and the molar ratio of P to Zn **(I)** during the grain development in 2009. The error bars represent the standard error of the mean (*n* = 4). ns indicates no significant difference. *, **, and *** indicate significant difference at *P* < 0.05, < 0.01, and < 0.001, respectively.

Although we observed an increase in DW of grain toward maize maturity, the highest grain concentrations of N, P, and Zn were found at the beginning of grain development (around 5–10 DAS). Thereafter, grain concentrations of N, P, and Zn rapidly declined between 10 to 15 DAS, and then further declined (less sharply) until the late milk stage (from 15 to 35 DAS), before remaining relatively constant until maturity (**Figures 5A, B, D, F**). The significant and positive effects of N supply on DW, and N, P, and Zn accumulation in grain were found during grain development stages with the effect being more pronounced towards grain development. Compared to the N0 treatment, N supply (N1 and N2 treatments) significantly increased the grain DW. However, there was a non-significant difference in grain DW between N1 and N2 treatments, with the exception that there was a significant increase at 10 DAS by increasing the N supply from N1 to N2 (**Figure 5A**). Similar results were also found for N, P, and Zn accumulation in grain, with the exception that there was a significant increase in grain Zn accumulation at 15, 30, and 35 DAS by increasing the N supply from N1 to N2 (**Figures 5C, E, G**). N supply generally tended to increase grain N and Zn concentrations during all stages of grain development with an exception at 10 DAS, compared with N0 treatment (**Figures 5B, D**). In addition, irrespective of N supply, the ratio of grain Zn to N concentration tended to decrease between 5 and 15 DAS and between 30 and 47 DAS, whereas the ratio of grain Zn to N concentration increased between 15 and 30 DAS and between 47 and 54 DAS (**Figure 5H**). Compared with the N0 treatment, the grain Zn/N concentration ratios were significantly higher than those with N1 and/or N2 treatments at 10, 15, 20, and 54 DAS (**Figure 5H**). However, compared with N0 treatment, N supply generally decreased the grain P concentration and resulted in significantly lower grain P concentration at five (10, 15, 25, 35, and 47 DAS) out of the recorded 10 stages (**Figure 5F**). Compared with N0 treatment, N supply also significantly decreased the molar ratio of P to Zn, but there was a non-significant difference between N1 and N2 treatments during all stages of grain development (**Figure 5I**).

### Shoot Zn Uptake and Zn Remobilization During the Grain-Filling Period

As shown in **Table 3**, during the grain-filling period, post-silking Zn uptake was 0.8, 1.6, and 2.3 mg plant^–1^ in 2009 and 1.0, 1.8, and 2.3 mg plant^–1^ in 2010 according to N0, N1, and N2 treatments, respectively. These values correspond to 31.4% to 44.7% and 34.0% to 42.2% of total shoot Zn accumulation at maturity. By contrast, 55.3% to 68.6% and 57.8% to 66.0% of the shoot Zn was accumulated by anthesis in 2009 and in 2010, respectively. Zn remobilization into grain ranged from 0.08 to 0.62 mg plant^–1^ in 2009 and from 0.03 to 0.46 mg plant^–1^ in 2010 among the three N rates (N0, N1, and N2, respectively). Straw Zn remobilization efficiency was greater in N0 (29.7% and 17.9%) than N2 (2.7% and 0.9%) in 2009 and 2010, respectively. Among the different vegetative tissues (leaves, stem, and cob), stem Zn accumulation decreased and stem Zn remobilization efficiency was 40.6%, 27.3%, and 14.2% according to N0, N1, and N2 treatments during the grain-filling period in 2010 (**Figure S3** and **Table S2**). The percentage of grain Zn provided by remobilization of the pre-silking Zn accumulation, was 3.3% (N2) and 39.4% (N0) in 2009, and 1.2% (N2) and 25.5% (N0) in 2010. This relationship reveals a progressively decreasing trend with increasing N rate. By contrast, 60.6% (N0) and 98.8% (N2) of grain Zn at maturity was provided by post-anthesis Zn uptake, and this relationship reveals an increasing trend with increasing N supply. Furthermore, post-anthesis Zn uptake was significantly correlated with post-anthesis N uptake (*r* = 0.775***) and grain Zn concentration (*r* = 0.738***) (**Table 2**).

**Table 3:** Straw remobilization ratios and contributions of remobilization of pre-silking Zn stores and post-silking shoot Zn uptake to grain Zn accumulation of summer maize as affected by N application rates in 2009 and in 2010.

Year	Parameter	N treatment		
		N0	N1	N2
2009	Post-anthesis Zn uptake^a^ (mg plant^–1^)	0.8	1.6	2.3
	Percentage of pre-anthesis Zn accumulation (%)	68.6	66.3	55.3
	Percentage of post-anthesis Zn accumulation (%)	31.4	33.7	44.7
	Zn remobilization to the grain^b^ (mg plant^–1^)	0.52	0.62	0.08
	Zn remobilization efficiency (%)	29.7	19.5	2.7
	Share of grain Zn provided by remobilization (%)	39.4	27.8	3.3
	Share of post-anthesis shoot Zn uptake (%)	60.6	72.2	96.7
2010	Post-anthesis Zn uptake^a^ (mg plant^–1^)	1.0	1.8	2.3
	Percentage of pre-anthesis Zn accumulation (%)	65.7	66.0	57.8
	Percentage of post-anthesis Zn accumulation (%)	34.3	34.0	42.2
	Zn remobilization to the grain^b^ (mg plant^–1^)	0.33	0.46	0.03
	Zn remobilization efficiency (%)	17.9	13.3	0.9
	Share of grain Zn provided by remobilization (%)	25.5	20.5	1.2
	Share of post-anthesis shoot Zn uptake (%)	74.5	79.5	98.8

^a^ Assume: ∼100% of post-silking Zn uptake from soil absorbed by roots.

^b^ Assume: ∼100% of Zn remobilized from the straw is translocated to grains.

## Discussion

In this study, soil DTPA-extractable Zn concentrations before sowing were 2.3 and 2.8 mg kg^–1^ in 2009 and 2010, respectively. This means that the soil used in this experiment can be classified as a high Zn one according to the standards (2.1–5.0 mg kg^–1^) for calcareous soil proposed by Liu et al. (1982). Under this condition, N application had effects not only on the growth and N nutrition but also Zn nutrition of maize. Dry weight and N and Zn concentration of grain and straw were significantly increased with the increasing N rate from null N to optimal N supply (150 kg N ha^−1^ in 2009 and 105 kg N ha^−1^ in 2010), but these values were not significantly increased with the further increasing N rate from optimal N supply (105/150 kg N ha^−1^) to traditional N application (250 kg N ha^–1^) in both cropping seasons (**Table 1**). However, the traditional N application resulted in a substantial improvement in the soil NO_3_^–^N contents at deep depths (30–60 and 60–90 cm) compared with the optimal N supply (**Figure S2**). These results indicate that optimized N management, by decreasing N input (40.0%–58.0% lower N input), is already sufficient to maintain plant growth and grain N and Zn nutrition of maize with lower environmental costs under our experimental conditions.

Maize grain Zn concentration was increased by 14.6–26.9% with the N application compared with null N application during the two years (**Table 1**). The extent of the increase with N supply was lower than 41.0%–73.9% with the increasing Zn application rates from 25 to 150 kg ZnSO_4._7H_2_O ha^–1^, compared with null Zn application average across the second and third years (calculated from the results of Liu et al., 2016). This finding is because the latter significantly improved available soil DTPA-Zn concentration to thereafter favor root Zn uptake and its allocation to grain. However, increasing N rates resulted in a 40% of reduction in Zn concentration of stover and cob and 20% of reduction in grain Zn concentration of maize possibly due to a marginal Zn deficiency in soil (Miner et al., 2018).Therefore, optimal N management, together with soil application of Zn fertilizer is a more practical and effective means to achieve simultaneous increases in both grain yield and Zn concentration, considering the difficulty of operating the foliar sprayer at the late growth stages of maize (Liu et al., 2016). In agreement with our previous results (Xue et al., 2014b), the positive effects of the N supply on Zn concentration of shoot tissues in maize were also found (**Table 1**, **Figure 3**), although these results are less pronounced, in comparison with wheat (Kutman et al., 2010; Xue et al., 2012; Xue et al., 2014a). Therefore, increasing N supply could also improve shoot Zn accumulation by maize. For example, compared to the null N treatment in 2010, the relative increases in shoot Zn accumulation at R1 stage with optimal and traditional treatments were 89.7% and 74.2%, respectively, whereas their corresponding increases in shoot dry matter were 59.0% and 65.3% (**Figures 1** and **3**). Similar results were also found at almost all other growing stages in both cropping seasons (**Figures 1** and **3**). By increasing N supply, shoot Zn accumulation increased more than shoot DW, suggesting a synergistic effect. Concurrently, Riedell (2010) reported that increasing N supply triggered a synergistic response between shoot DW accumulation and N, calcium, and manganese, but a diluted response between shoot DW accumulation, P, and potassium of maize. Recent study showed that fertilizer N application synergistically affected the plant copper nutrition, whereas there was a dilution effect on plant Zn and magnesium concentrations of maize (Ma and Zheng, 2018). Our results further showed that shoot Zn concentration and accumulation of maize tended to decrease if the soil available Nmin at 0-90 cm was more than 176 kg ha^–1^ at R1 stage and 344 kg N ha^–1^ at R6 stage, with excessive N supply (**Figure 4**).

Zinc remobilization efficiency of maize only ranged from 0.9% to 29.7% whereas that of wheat reached up to 50% to 81% (Xue et al., 2012). Consequently, 60.6% to 98.8% of grain Zn content of maize was provided by continued post-silking Zn uptake (**Table 3**), whereas 67% to 100% of that of wheat was provided by Zn remobilization from pre-anthesis Zn accumulation under a wheat-maize cropping system (Xue et al., 2012). Furthermore, post-anthesis Zn uptake was significantly correlated with grain Zn concentration (*r* = 0.738***) (**Table 2**). These results show that, in contrast with wheat (Xue et al., 2012; Zhang et al., 2015b), vegetative tissues of maize are only minor Zn sources, whereas post-silking shoot Zn uptake from the soil during grain filing was the major source for grain Zn accumulation of maize, in agreement with other studies focusing on maize (Grzebisz et al., 2008; Chen et al., 2016). Kutman et al. (2010) suggested that Zn, or any other mineral taken up by the shoot during grain filling, can either be directly translocated to the grain or first reach a source tissue, from which it is re-translocated to the grain. Therefore, the grain Zn accumulation of maize derived from either and/or both of these pathways described above need to be further investigated.

It is not surprising that the grain Zn accumulation of maize and wheat is derived from different sources under filed conditions. In the North Plain of China, annual precipitation is 500 to 700 mm with approximately 70% of rainfall occurring during the maize growing season (Zhang et al., 2013), which was favorable to post-silking Zn uptake of maize during grain-filling stage. Therefore, to gain higher grain yield and avoid Zn deficiency in maize, more Zn and a season-long supply of Zn is necessary, especially during the V12 to R1 growth stages when the highest Zn accumulation rate occurs (Xue et al., 2014b). However, the North Plain of China has mainly calcareous soils with limited soil moisture and is exposed to continental heat, dry winds, high temperatures, and water deficiency during grain filling of wheat (Yang et al., 2000). Consequently, a continued root uptake was hampered, and grain Zn relied mostly on export of Zn from the vegetative parts of wheat during grain formation. These results suggest that the significant contributions of pre- and/or post-silking Zn uptake to grain Zn accumulation depended greatly on cereal species and especially the post-anthesis availability of Zn in the growth medium for uptake as well as the N nutritional status (Waters et al., 2009; Kutman et al., 2012).

As reported previously for wheat and maize (Grzebisz et al., 2008; Kutman et al., 2011), our results showed that stems were an important Zn store that could be utilized during the grain-filling stage, but remobilization efficiency of stem Zn was negatively affected by increasing N supply (**Table S2**). By contrast, post-silking Zn uptake (0.8–2.3 mg plant^–1^) and its substantial contributions to grain Zn accumulation (60.6% to 98.8%) were progressively enhanced with the increasing N supply (**Table 3**). Furthermore, a significant positive relationship was found between post-silking Zn uptake and post-anthesis N uptake (*r* = 0.775***). The positive impacts of N on root Zn uptake and root-to-shoot translocation could possibly be related to N-stimulated abundance of root Zn uptake transporters, including ZIPs, such as IRT1 and other unknown proteins, N enhanced-activities of transporter proteins contributing to xylem loading, or nitrogenous compounds facilitating Zn transport in plants, such as nicotianamine (NA) and deoxymugineic acid (DMA) (Erenoglu et al., 2011; Nozoye, 2018). Diaz-Benito et al. (2018) recently reported that increasing DMA alone led to iron enrichment in the embryo and endosperm, whereas increasing DMA in combination with NA led to iron and Zn enrichment in both tissues of rice.

Increasing N supply may delay senescence and thereby extended the grain-filling period to allow more nutrient transport (such as N and Zn) into the grain (Yang and Zhang, 2006; Bogard et al., 2010). Kutman et al. (2012) showed that Zn remobilized from pre-anthesis reserves in the straw could provide almost 100% of the grain Zn if the plants failed to take up Zn from the root system during grain development. However, up to 100% of the grain Zn could be attributed to Zn uptake during the seed-filling stage if the Zn uptake continued and a high N supply improved the uptake by delaying senescence and extending the stay-green period in a hydroponically-grown wheat. Peng et al. (2012) reported that the delayed root senescence with sufficient N supply compared with N deficiency may accelerate robust nutrient (such as Zn and N) uptake early in the growing season, and translocation from roots to reproductive organs later in the season. Another possible explanation is that increased N supply may create a larger sink in the grain for Zn accumulation (Morgounov et al., 2007; Peleg et al., 2008; Zhao et al., 2009) because grain Zn concentration positively associated with grain N concentration (r = 0.752***) and post-silking N uptake (r = 0.695***). Likewise, Horvatić et al. (1998) reported that Zn correlated positively and significantly with the total N but significantly and negatively with PA in maturing grains of maize. Furthermore, Zn fertilization application significantly reduced grain PA and increased grain Zn and protein concentrations of maize (Imran and Rehim, 2016). This present study further showed that both grain P concentration and the molar ratio of P to Zn were decreased, whereas grain Zn concentration was increased with N supply during grain maturation, suggesting an increasing Zn bioavailability in maize grain.

## Conclusion

N application significantly affected the dynamics of Zn accumulation in plants and grain together with the pre-silking and post-silking Zn uptake and their contributions to grain Zn accumulation of maize in the field. Zinc accumulation in maize grain mainly originated from post-silking Zn uptake and the optimal N management ensures better shoot Zn nutrition thereafter to contribute to the post-silking Zn uptake. The substantial contribution of post-silking Zn uptake to grain Zn accumulation maintains higher grain Zn availability by decreasing the grain P concentration and the ratio of P to Zn, resulting in grain that is better for human nutrition. Therefore, the optimal N management, together with soil application of Zn fertilizer, is a more practical and effective strategy for simultaneously increasing both grain yield and Zn concentration, especially considering the difficulty of operating the foliar sprayer at the late growth stage of maize.

## Author Contributions

C-QZ and X-PC conceived and designed the experiments. Y-FX, S-CY, D-YL, and WZ performed the experiments. C-QZ and Y-FX analyzed the data and wrote the paper. All the authors read and approved the final manuscript.

## Funding

This research was supported by 973 project (2015CB 150402), the China Agriculture Research System (CARS-02), the National Natural Science Foundation of China (31272252, 31702001), the National Key Research and Development Program of China (2018YFD0200603) and the High-Level Talents and Innovative Team Recruitment Program of the Shandong Academy of Agricultural Sciences.

## Conflict of Interest

The authors declarthat the research was conducted in the absence of any commercial or financial relationships that could be construed as a potential conflict of interest.
