# Supplementary material for: Dynamic Zinc Accumulation and Contributions of Pre- and/or Post-Silking Zinc Uptake to Grain Zinc of Maize as Affected by Nitrogen Supply
Source: Front Plant Sci. 2019 Oct 3;10:1203. doi: 10.3389/fpls.2019.01203 (PMC6785940; doi:10.3389/fpls.2019.01203)
Supplement: Supplementary file 1 [file Image_1.pdf]

SUPPLEMENTARY MATERIALS

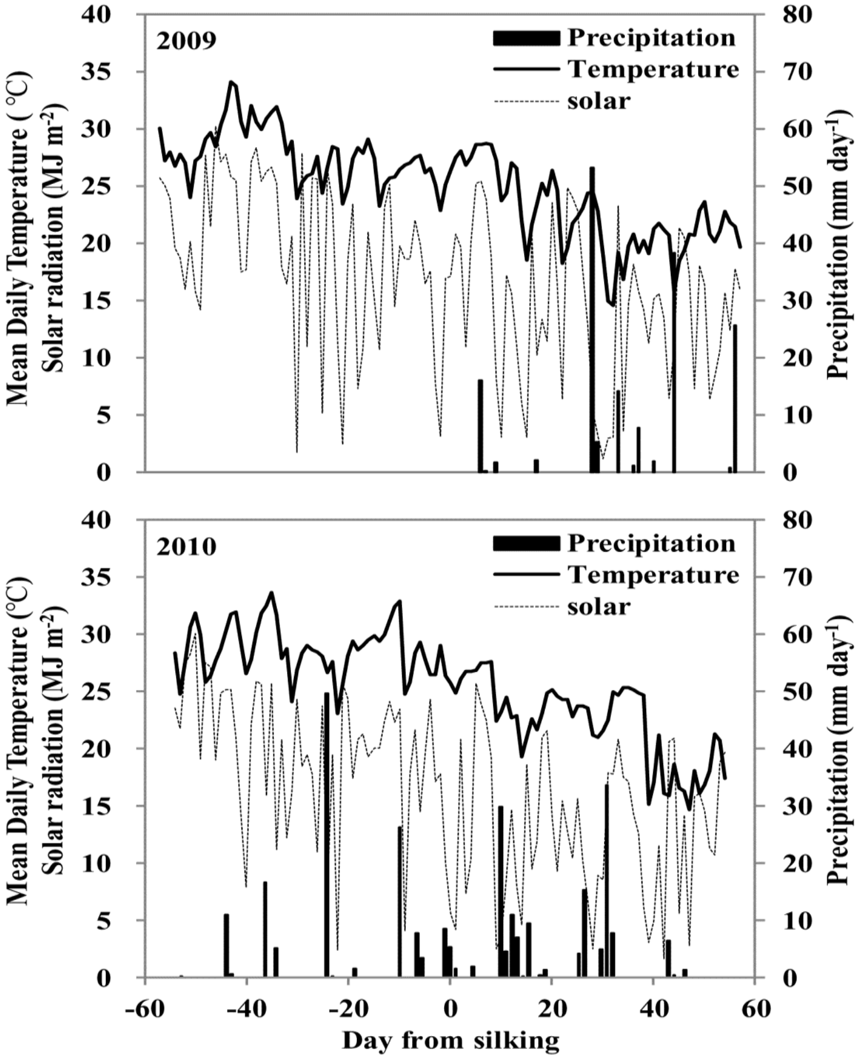

FIGURE S1. Daily mean temperature, solar radiation and precipitation during the maize growing seasons in 2009 and 2010 at Quzhou (QZ) County (36.9 °N, 115.0 °E), Hebei province, China.

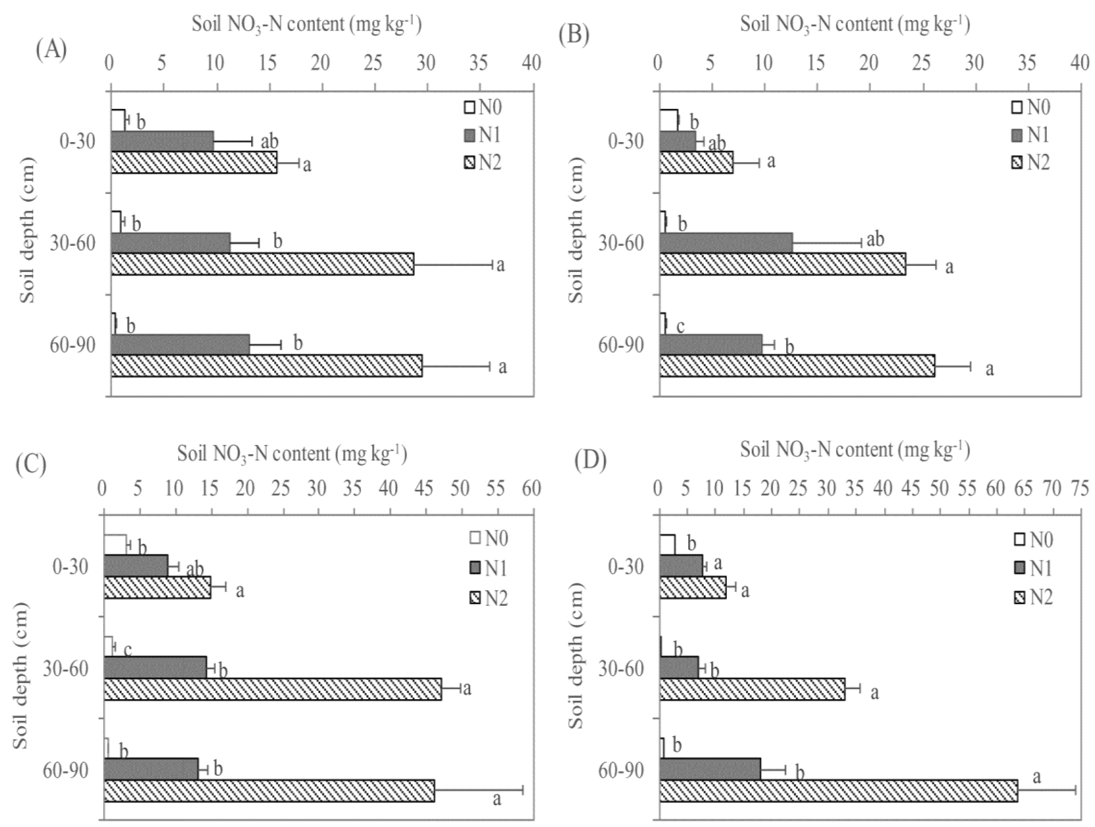

FIGURE S2. The NO_3^--N contents at different soil layers (0-30, 30-60 and 60-90 cm) at R1 and R6 growth stages, respectively, in 2009 (A, C) and 2010 (B, D)

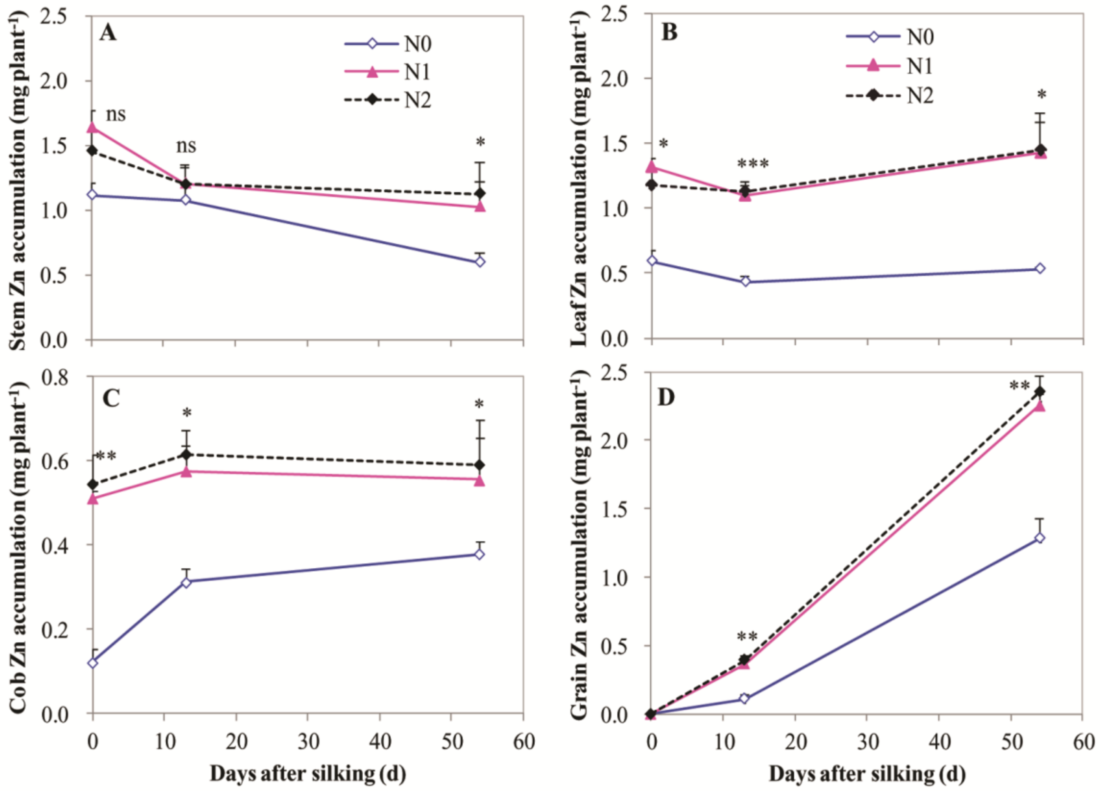

FIGURE S3. Zn accumulation in maize shoot parts including stems (A), leaves (B), cobs (C) and grains (D) at R1, R3 and R6 stages as affected by different N treatments in 2010. The bars represent the standard error of the mean (n = 3).

TABLE S1 N target value, soil nitrate-N content in the root layers, and N application rates for different growth stages and years.

Growth stage	N target value (kg ha^{-1})	Soil nitrate-N (kg ha^{-1})		N application rate (kg ha^{-1})	
		2009	2010	2009	2010
Before sowing	–	23.5*	43.0*	45	45
6-leaf stage	120	119.5**	109.8**	30	30
10-leaf stage	190	118.9**	173.1***	75	30

* Soil nitrate-N content in the top 30 cm before sowing

** Soil nitrate-N content in the top 60 cm at the 6-leaf stage

*** Soil nitrate-N content in the top 90 cm at the 10-leaf stage

TABLE S2 Percentage of grain Zn at maturity provided by remobilization of pre-silking Zn stores in vegetative tissues (leaves, stems and cob) and post-silking Zn uptake as affected by different N application rates (N0: zero N application; N1: 105 kg N ha^{-1}; N2: 250 kg N ha^{-1}) in 2010.

**Parameter**	**N treatment**		
	**N0**	**N1**	**N2**
Remobilization of pre-silking Zn (%)			
Leaves	4.8	-4.8	-11.1
Stems	40.6	27.3	14.2
Cob	-20.0	-1.9	-2.0
Straw	25.5	20.5	1.2
Post-silking shoot Zn uptake (%)	74.5	79.5	98.8
